# *CHIP* modulates APP‐induced autophagy‐dependent pathological symptoms in *Drosophila*

**DOI:** 10.1111/acel.13070

**Published:** 2019-11-28

**Authors:** Luming Zhuang, Fei Peng, Yuanyuan Huang, Wenzhe Li, Jiuhong Huang, Yunqiang Chu, Pu Ren, Ying Sun, Yan Zhang, Elleen Xue, Xiaowei Guo, Xiafeng Shen, Lei Xue

**Affiliations:** ^1^ The First Rehabilitation Hospital of Shanghai Shanghai Key Laboratory of Signaling and Diseases Research School of Life Science and Technology Tongji University Shanghai China; ^2^ International Academy of Targeted Therapeutics and Innovation Chongqing University of Arts and Sciences Chongqing China; ^3^ Blair Academy Blairstown NJ USA

**Keywords:** Alzheimer’s disease, APP, autophagy, Aβ, *CHIP*

## Abstract

Dysregulation of autophagy is associated with the neurodegenerative processes in Alzheimer's disease (AD), yet it remains controversial whether autophagy is a cause or consequence of AD. We have previously expressed the full‐length human APP in *Drosophila* and established a fly AD model that exhibits multiple AD‐like symptoms. Here we report that depletion of *CHIP* effectively palliated APP‐induced pathological symptoms, including morphological, behavioral, and cognitive defects. Mechanistically, CHIP is required for APP‐induced autophagy dysfunction, which promotes Aβ production via increased expression of *BACE* and *Psn*. Our findings suggest that aberrant autophagy is not only a consequence of abnormal APP activity, but also contributes to dysregulated APP metabolism and subsequent AD pathogenesis.

## Graphical abstract

CHIP modulates APP‐induced Aβ production and pathological symptoms. CHIP promotes APP‐induced aberrant autophagy. Autophagy contributes to APP‐induced pathological symptoms. Autophagy regulates Aβ production and amyloid deposits.

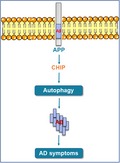

## 1 INTRODUCTION

Alzheimer's disease (AD) is the most common chronic neurodegenerative disease among senior individuals. AD is clinically characterized by progressive decline in memory (Hardy, 2006), and histologically characterized by neuronal loss (Gunawardena & Goldstein, 2001), dystrophic neurites (Glenner, 1989), neurofibrillary tangles of hyperphosphorylated Tau (Goedert, Wischik, Wischik, Crowther, Walker, & Klug, 1988), and extracellular amyloid plaques (Selkoe, Abraham, Abraham, Podlisny, & Duffy, 1986). Amyloid plaques largely consist of the amyloid‐β (Aβ) peptide, which is produced from the amyloid precursor protein (APP) by sequential cleavage of β‐ and γ‐secretases (Cupers et al., 2001). APP encodes a type I integral membrane protein with three major isoforms, among which the 695‐residue isoform is the most common in neurons (Nhan, Chiang, Chiang, & Koo, 2015), and has been implicated in the pathogenesis of AD (Fernandez‐Funez, Mena, & Rincon‐Limas, 2015). Genetic studies of familial AD (FAD) have identified mutations in APP and its associated genes in the early‐onset AD (Bertram & Tanzi, 2008), while many risk factors or genes associated with the late‐onset AD also affect APP metabolism and Aβ production (Wen et al., 2013).

Autophagy is an evolutionarily conserved catabolic process leading to clearance of proteins and dysfunctional organelles, and is induced mostly by starvation, oxidative stress, and neuronal toxicity (Levine & Kroemer, 2008). In the brain, autophagic activity is maintained at low levels even with nutrient starvation (Kuma et al., 2004). Yet, overactivated autophagy is observed in AD patients, with the marked accumulation of autophagosomes and the aggregation of autophagic vacuoles in dystrophic neurites of AD brains (Yu et al., 2004). The same phenotype is also observed in AD‐mouse models and neuronal cells treated with Aβ peptide (Yang et al., 2011). There is an emerging agreement that autophagy defects likely contribute to the neurodegenerative processes in AD (Zare‐Shahabadi, Masliah, Masliah, Johnson, & Rezaei, 2015). However, little is known about the mechanism by which autophagy deficits are regulated in AD.

The carboxyl‐terminus of Hsc70‐interacting protein (CHIP) is a U‐box type chaperone associated E3 ligase that contains the N‐terminal tetratricopeptide repeats domain involved in the protein–protein interaction and the C‐terminal U‐box domain responsible for catalyzing the transfer of ubiquitin to substrate proteins (Paul & Ghosh, 2014). CHIP has been reported to play multiple functions, such as protein degradation (Al‐Ramahi et al., 2006), signal transduction (Tawo et al., 2017), apoptosis (Hwang, Cho, Cho, & Ahn, 2018), and autophagy (Arndt et al., 2010; Ferreira et al., 2013; Guo et al., 2015). However, the role of CHIP in AD pathogenesis remains unclear.

In this study, we found that CHIP is indispensable for APP‐induced AD‐like symptoms in *Drosophila*, which include wing expansion defect, photoreceptor degeneration, DA neuron loss, locomotor disability, lifespan shortening, choice, and learning deficits. Mechanistically, CHIP promotes APP‐induced aberrant autophagy, which contributes to Aβ production through transcriptional upregulation of *BACE1* and *Psn*, and subsequent AD‐like neurodegeneration.

## 2 RESULTS

### 2.1 *CHIP* depletion palliates APP‐induced wing expansion defect

**Figure 1:**
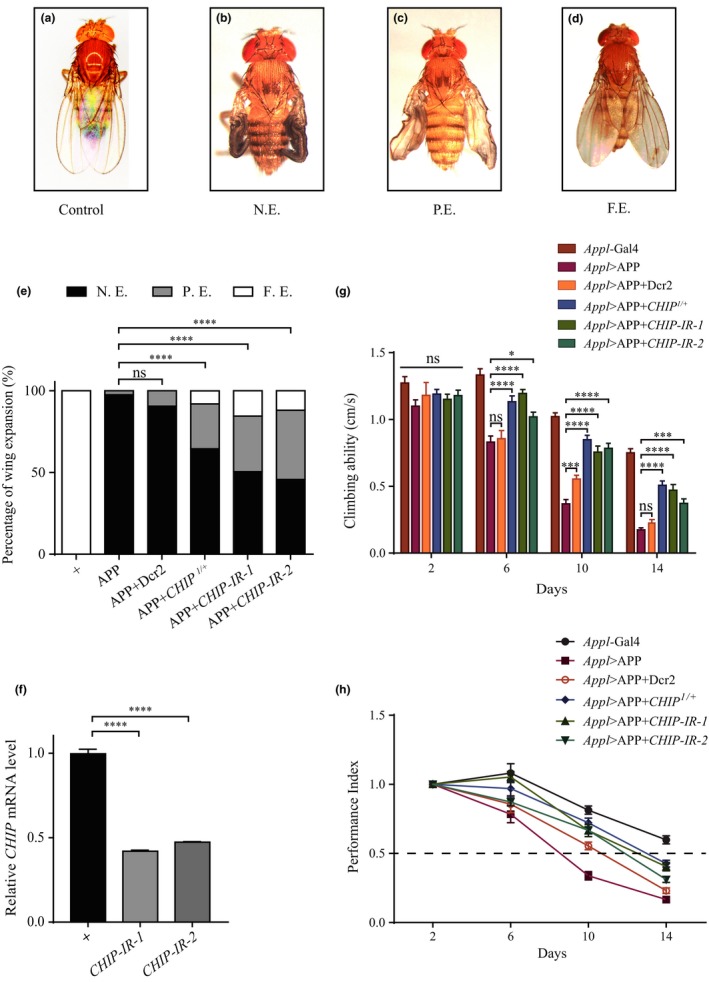
*CHIP* depletion palliates APP‐induced wing expansion defect and adult‐specific locomotor deficits. (a–d) Images showing varying degree of wing expansion phenotypes in adult female flies raised at 25℃ (abbreviation: N.E., no expansion; P.E., partial expansion; F.E., full expansion). Compared with the *Appl*‐Gal4 controls (a), overexpression of APP driven by *Appl*‐Gal4 produces an infant wing phenotype (no expansion, b), which is suppressed partially (c) or fully (d) in heterozygous *CHIP^1^* mutants, or by RNAi‐mediated down‐regulation of *CHIP*, but remained unaffected by expressing *UAS*‐Dcr2. (e) Histogram showing the percentage of adult wing phenotypes in different genotypes. The number of female flies tested for each genotype is *n* > 100. (f) Histogram showing the knockdown efficiencies of two independent *UAS‐CHIP‐RNAi* lines measured by qRT–PCR. Data were obtained from three independent biological replicates. (g, h) Histograms and line charts showing longitudinal activity of the indicated genotypes at different time points. (g) Compared with the controls, adult‐specific expression of APP has no discernable effect on the climbing ability of 2‐day‐old flies, but displays an age‐dependent decline of climbing ability after day 6. Decrease of *CHIP* significantly alleviates APP‐induced locomotor deficits. (h) *Appl*‐Gal4 controls reach 50% climbing index after day 16. *Appl* > APP flies reach 50% climbing index after day 8, which is partially restored to days 12–13 by depletion of *CHIP*, but remained unaffected by expressing *UAS*‐Dcr2. All values are shown as mean ± *SEM*. *p < .05, ***p < .001, ****p < .0001, ns, not significant. More than 200 female flies were tested per genotype

*Drosophila* wing expansion is governed by the neurohormone bursicon (burs), and dysfunction of bursicon‐expressing neurons in the CNS results in wing expansion defect and produces an infant wing phenotype (Peabody et al., 2008). Pan‐neural overexpression of APPL, the *Drosophila* homologue of APP, driven by *Appl‐*Gal4 (*Appl* > APPL), disrupts neuroendocrine function and generates a similar infantile phenotype (Torroja, Chu, Chu, Kotovsky, & White, 1999), which is recapitulated by expressing human APP (*Appl* > APP; Figure 1b; Peng et al., 2015). Thus, this pathological function of APP/APPL has been evolutionary conserved from fly to human, and factors that regulate this function are likely conserved as well. To identify additional genes that modulate APP’s pathological functions, we have previously performed a genetic screen for dominant modifiers of APP‐induced wing expansion defect (Peng et al., 2015). We found that *Appl* > APP‐induced wing defect was effectively suppressed in heterozygous *CHIP^1^* mutants (28% partial expansion and 8% full expansion, Figure 1c–e). Consistently, APP‐induced infantile wing phenotype was suppressed by expressing two independent *CHIP* RNAi (*CHIP‐IR‐1*, 34% partial expansion and 15% full expansion; *CHIP‐IR‐2*, 42% partial expansion and 11% full expansion, Figure 1e), but remained unaffected by the expression of Dcr2 serving as a negative control (Figure 1e). Depletion of *CHIP* by itself did not produce any discernible wing phenotype (Figure S1a–d). The knockdown efficiencies of the two *CHIP‐RNAi* lines were verified by quantitative reverse transcription–polymerase chain reaction (qRT–PCR; Figure 1f). On the other hand, *CHIP* transcription in 3rd instar larval brains and adult heads was not significantly altered by APP overexpression (Figure S2). Since *Appl* is located on the X chromosome, *Appl* > APP males are lethal due to the dosage compensation effect, only females are used in this and following experiments, except in the courtship choice and learning assay where *fru* > APP and *elav* > APP males were utilized, respectively. Overall, these results suggest that *CHIP* reduction palliates APP‐induced wing expansion defect.

### 2.2 *CHIP* depletion alleviates APP‐induced locomotor deficit

To verify the physiological benefits of *CHIP* depletion on APP’s pathological functions, we next examined the locomotor performance of adult flies as a surrogate functional assay for APP‐induced neurotoxicity (Iijima et al., 2004). For AD is an age‐related disease, to overcome the developmental defects and investigate the pathological functions of APP in aging adults, we took advantage of the temperature dependence of Gal4 activity (Duffy, 2002). To this end, APP expression was restricted throughout development at 17℃ due to the minimal Gal4 activity and was activated specifically in adulthood by shifting to 29℃ after eclosion (Figure S3). As expected, such flies displayed normal wings and climbing ability that was indistinguishable from that of *Appl*‐Gal4 controls at 2 days after eclosion (Figure 1g). However, at day 6, *Appl* > APP flies showed a dramatically reduced climbing ability (0.83 cm/s), as compared with age‐matched controls (1.33 cm/s; Figure 1g). APP‐induced locomotor defect was significantly suppressed in heterozygous *CHIP^1^* mutants (1.13 cm/s), or by expressing two *CHIP‐IR* lines (1.19 and 1.02 cm/s). At days 10 and 14, the control flies displayed a gradual reduction of climbing ability, indicating an age‐dependent locomotor decline, which was accelerated by APP expression (Figure 1g). Again, decrease of *CHIP* was able to suppress APP‐induced locomotor deterioration (Figure 1g). As a control, decrease of *CHIP* alone did not alter the climbing ability (Figure S1e).

To directly evaluate the locomotor decline, we defined a performance index (PI) by comparing the climbing velocity between aged and young (2‐day‐old) flies: PI = velocity_aged_/velocity_young_ (Peng et al., 2015). We found that *Appl*‐Gal4 controls displayed a gentle decline with aging (Figure 1h, reached 50% PI after day 16). APP‐expressing flies revealed a more obvious and drastic decline (Figure 1h, reached 50% PI by day 8), which was strikingly suppressed by down‐regulation of *CHIP* (Figure 1h, reached 50% PI between day 12 and day 13). Together, these observations indicate that decrease of *CHIP* alleviates APP‐induced, age‐dependent locomotor deficit of adult flies.

### 2.3 Down‐regulation of *CHIP* suppresses APP‐induced toxicity in eye development

**Figure 2:**
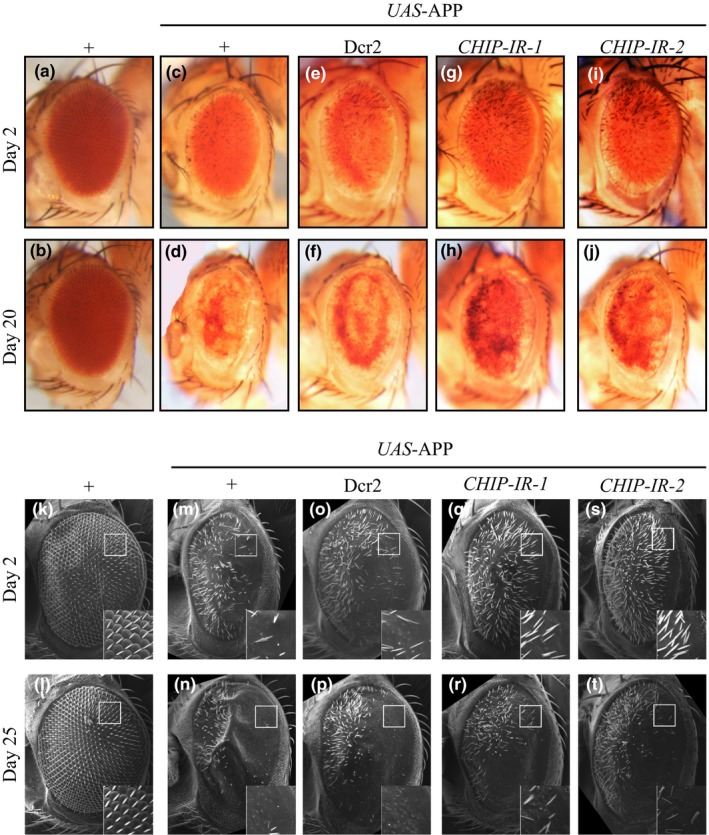
Down‐regulation of *CHIP* suppresses APP‐induced toxicity in eye development. (a‐j) Optical microscopic images showing eye phenotypes in 2‐ and 20‐day‐old female flies. *GMR*‐Gal4 controls display a highly ordered ommatidial lattice (a, b). Overexpression of APP driven by *GMR*‐Gal4 causes roughened and depigmented eyes at day 2 (c), which becomes increasingly obvious in 20‐day‐old females (d). APP‐induced eye phenotypes are suppressed by depletion of *CHIP* (g–j), but not by expression of Dcr2 (e, f). (k–t) Images showing *SEM* of eyes from 2‐ and 25‐day‐old females. Compared with the controls (k, l), APP expression induces the loss of interommatidial bristles at day 2 (m), which is exacerbated at day 25 (n). The defect is suppressed by depletion of *CHIP* (q–t), but remains unaffected by expressing Dcr2 (o, p). Female flies were raised at 25°C and shifted to 29°C after eclosion, *n* > 15 per genotype

*Drosophila* eyes have been widely used to express human neurotoxic proteins to approximate neurodegenerative diseases (Burr, Tsou, Tsou, Ristic, & Todi, 2014). Co‐expression of human APP and BACE1 in fly compound eyes is able to induce age‐dependent neurodegeneration of the photoreceptor cells (Greeve et al., 2004). Since *Drosophila* also encodes a protein with β‐secretase activity, we wonder whether APP expression is sufficient to trigger neurodegeneration in fly eyes. To this end, we mobilized the *UAS*‐APP transgene by the Δ2–3 transposase located at 99B with standard fly genetics and obtained a stronger line located on the 2nd chromosome. We found the *GMR*‐Gal4 controls showed a normal, well‐organized, and smooth external eye surface on day 2 after eclosion (Figure 2a,k), and displayed no obvious morphological changes with age (Figure 2b,l). However, expression of APP (*GMR* > APP) not only resulted in small, rough, and depigmented eyes, but also induced the loss of interommatidial bristles (Figure 2c,m), which became more drastic in aged flies (Figure 2d,n). These phenotypes were suppressed by expressing *CHIP RNAi*, but not Dcr2 (Figure 2e–j,o–t), while *CHIP* depletion alone exhibited normal eye morphology (Figure S4). Together, these results suggest that *CHIP* is essential for APP‐induced age‐dependent photoreceptor degeneration.

### 2.4 Decrease of *CHIP* ameliorates APP‐induced age‐dependent DA neuron loss

**Figure 3:**
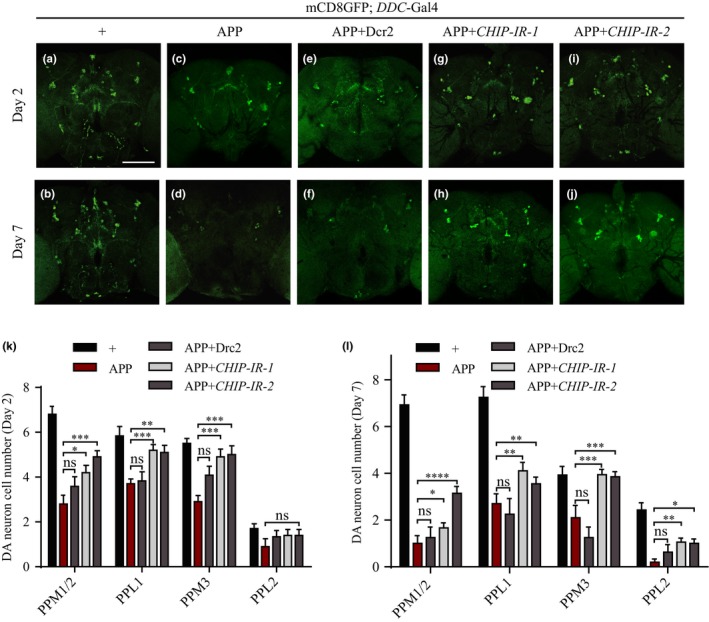
Decrease of *CHIP* ameliorates APP‐induced age‐dependent DA neuron loss. (a–j) Confocal images of DA neuron clusters in adult posterior brains. 2‐ and 7‐day‐old females’ brains were dissected. DA neurons are labeled with mCD8GFP (green) driven by *DDC*‐Gal4. Compared with the controls (a, b), overexpression of APP causes a reduction of cell number in most DA neuron clusters of 2‐day‐old adult brains (c), which becomes more obvious in 7‐day‐old brains (d). This age‐dependent DA neuron loss is ameliorated by depletion of *CHIP* (g–j), but not by expressing Dcr2 (e, f). (k, l) Statistical analysis showing the number of GFP‐positive cells in different DA neuron clusters. Values are shown as mean ± *SEM*. * < .05, **p < .01, ***p < .001, ****p < .0001, ns, not significant. *n* = 15 brains per genotype. Scale bars, 50 μm

For the progressive loss of neurons in the brain is a hallmark of AD (Hardy, 2006), we next sought to investigate the genetic interaction between *CHIP* and APP in this context. It has been reported that neurotransmitter dopamine (DA, released from DA neurons) modulates movement and cognition, and dopaminergic dysfunction plays a pathogenic role in cognitive decline symptoms of AD (Martorana & Koch, 2014). Consistently, progressive elimination of DA neurons has been observed in fly brains expressing APP (Bolshakova, Zhuk, Zhuk, Rodin, Kislik, & Sarantseva, 2014). To confirm whether expression of APP leads to age‐dependent DA neuron loss, we paid attention to four clusters of DA neurons in the central brain: paired posterior lateral 1 and 2 (PPL1 and PPL2); paired posterior medial 1 and 2 (PPM1/2) which are often grouped together because of their close proximity; and paired posterior medial 3 (PPM3). These neurons were labeled by a membrane‐bound GFP reporter driven by *DDC*‐Gal4 (Friggi‐Grelin et al., 2003). We found no significant difference in the number of DA neurons between control flies and APP‐expressing flies at 2 hr after eclosion (Figure S5). However, compared with the controls, APP‐expressing flies exhibited a significant loss of DA neurons in PPM1/2, PPL1, and PPM3 clusters at day 2, which was exacerbated at day 7 (Figure 3a–d,k,l). These results demonstrate APP‐induced DA neuron loss is an age‐dependent process, but not a developmental defect. APP‐induced neuron loss was considerably suppressed by *CHIP* depletion (Figure 3g–l), which by itself did not affect the number of DA neurons (Figure S6). As a negative control, expression of Dcr2 failed to suppress APP‐induced neuron loss (Figure 3e,f). Together, these data suggest that *CHIP* is necessary for APP‐induced age‐dependent DA neuron loss.

### 2.5 Down‐regulation of *CHIP* suppresses APP‐induced lifespan shortening

**Figure 4:**
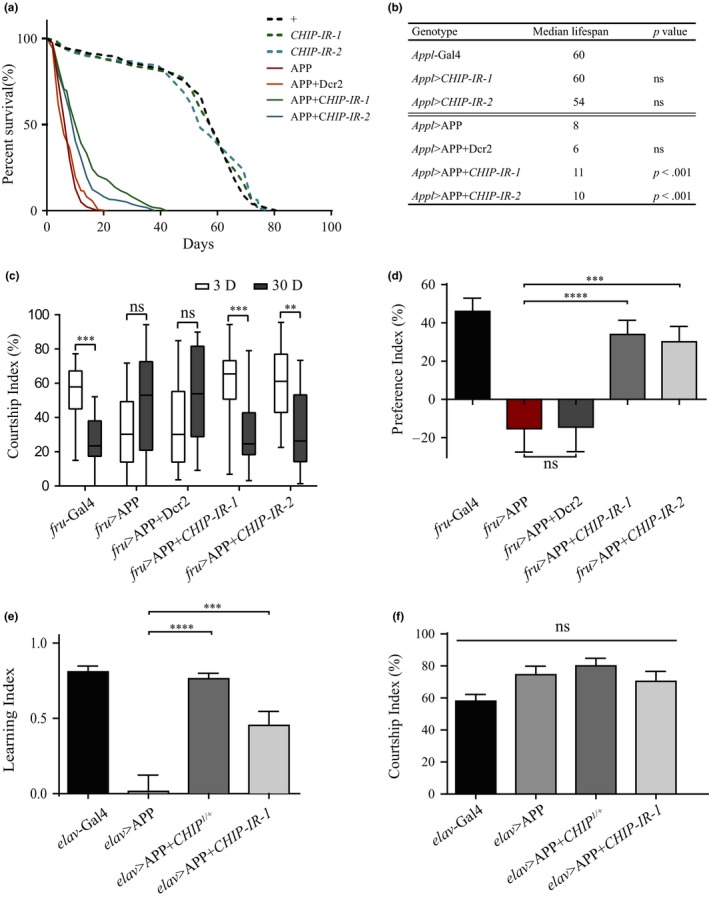
Down‐regulation of *CHIP* restores APP‐induced lifespan shortening, choice and learning defects. (a, b) Survival analysis of female flies with the indicated genotypes. Proportion of flies alive in percentage (live/total, %) was calculated every 2 days to monitor lifespan. (a) Compared with the controls, overexpression of APP driven by *Appl*‐Gal4 leads to a drastically shortened lifespan. Depletion of *CHIP* markedly suppresses the lifespan of APP flies. *UAS*‐Dcr2 as negative control had no effect on the APP‐induced lifespan shortening. (b) Summary of median lifespans is shown. *n* = 200 female flies per group. (c, d) Courtship index and preference index of 3‐day‐old naive males with indicated genotypes in choice assays. Compared with *fru*‐Gal4 control males, overexpression of APP driven by *fru*‐Gal4 abrogates the courtship preference for younger mates, which is restored by knocking down *CHIP*, but reminded unaffected by expressing *UAS*‐Dcr2. *n* > 30 males per genotype. (e) Learning index of 3‐day‐old naive males with indicated genotypes in the courtship suppression assay. Compared with *elav*‐Gal4 controls, overexpression of APP driven by *elav*‐Gal4 displays a learning impairment, which is improved by down‐regulation of *CHIP*. (f) Courtship index of naive males in the initial 10 min of the training phase in the courtship suppression assay, which implies that flies per genotypes have normal courtship ability. *n* > 30 males per genotype. Values are shown as mean ± *SEM*. **p < .01, ***p < .001, ****p < .0001, ns, not significant

We also monitored the longevity of the above flies, since AD seriously affects neurological integrity resulting in an increased risk of earlier death (Mhatre et al., 2014). The *Appl*‐Gal4 control flies lived up to ~80 days with a median lifespan of 60 days, whereas APP‐expressing flies exhibited a drastically reduced longevity with a median lifespan of 8 days, and a maximal lifespan of 18 days (Figure 4a,b). APP‐induced lifespan shortening was effectively suppressed by *CHIP* depletion, which by itself had no effect on the lifespan (Figure 4a,b). These results suggest that *CHIP* is indispensable for APP‐induced lifespan shortening.

### 2.6 Decrease of *CHIP* restores APP‐induced choice and learning defects

The above data indicate that decrease of *CHIP* salvages a diverse array of morphological and behavioral defects caused by APP overexpression. Mounting evidence suggests, despite its relative simplicity, the fly brain is able to drive sophisticated behaviors like decision‐making, learning, and memory. Since cognitive impairment is one of the major clinical characteristics of AD patients, we attempted to determine the role of *CHIP* in APP‐induced cognitive deficits. To this end, we first carried out the male courtship choice assays, in which male flies were provided with both younger and older virgin females simultaneously, and the courtship index (CI) was checked as previously described (Hu, Han, Han, Wang, & Xue, 2014). To accurately quantify the extent of males’ preference for younger or older females in courtship choice assay, we measured the preference index (PI) indicating a relative difference between males’ CI toward younger or older females (Hu et al., 2014). Consistent with the previous report (Hu et al., 2014), 3‐day‐old control males courted vigorously toward the younger females rather than the older ones (Figure 4c), indicating a courtship preference behavior for younger mates (Figure 4d). In contrast, 3‐day‐old males expressing APP driven by the courtship neuron‐specific *fruitless*‐Gal4 (Demir & Dickson, 2005) exhibited no preference for the younger mates (Figure 4c,d), suggesting a choice impairment induced by APP. APP‐induced courtship preference deficit was restored by depletion of *CHIP* (Figure 4c,d), which alone did not alter the preference behavior (Figure S7a). To rule out the possibility that APP‐induced choice impairment is a result of mobility decline, we checked the total CI and climbing ability of these males, and found no significant difference between control males and APP‐expressing males (Figure S7c,d). These results indicate that *CHIP* is involved in APP‐induced choice dysfunction.

Since AD is associated with impaired learning and memory, we wonder whether *CHIP* plays a role in APP‐induced learning deficit. To this end, we performed the courtship conditioning procedure (also termed as courtship suppression assay) wherein a male's courtship behavior is affected by experience with an unreceptive female (Siegel & Hall, 1979). Wild‐type naive males court virgin females persistently, displaying all defined courtship steps including orientation, tapping, wing extension, licking, and attempted copulation (Hall, 1994). However, after being paired with an unreceptive mated female for a certain period, a wild‐type naive male shows significantly decreased courtship activity, as the mated female displays an altered behavior and pheromone profile to reject the naive male constantly (Burnet, Connolly, Connolly, Kearney, & Cook, 1973).

To evaluate the learning ability, we performed the one‐hour courtship suppression training, measured the CI of the initial (CI_initial_) and final (CI_final_) 10 min, and defined the learning index as LI = (CI_initial_ − CI_final_)/CI_initial_. While 3‐day‐old *elav*‐Gal4 control naive males exhibited a robust learning ability, males with pan‐neuronal expression of APP (*elav* > APP) displayed a complete abolishment of learning ability, which was effectively restored by depletion of *CHIP* (Figure 4e), although decrease of *CHIP* alone had no effect on the males’ learning ability (Figure S7b). Noticeably, all males showed similar CI_initial_—courtship index in the initial 10 min of the training phase (Figure 4f), indicating that the normal courtship ability was not affected by APP expression. Overall, these results indicate that down‐regulation of *CHIP* restores APP‐induced learning defect.

### 2.7 Down‐regulation of *CHIP* suppresses APP‐induced autophagy

**Figure 5:**
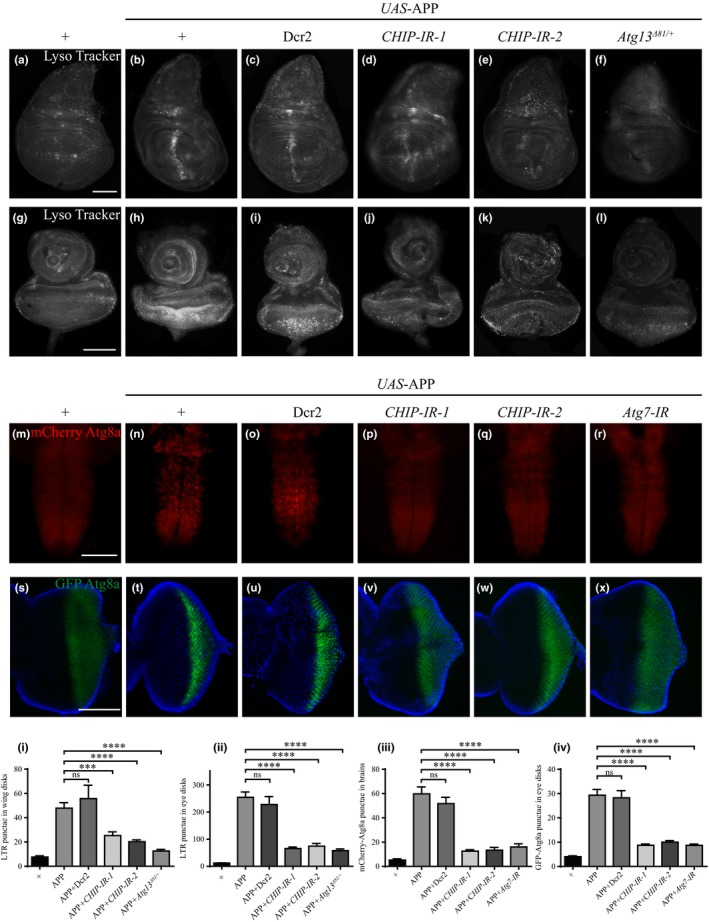
Depletion of *CHIP* impedes APP‐induced autophagy. Fluorescence microscopic images showing larval wing disks (a–f), eye disks (g–l, s–x), and ventral nerve cord (VNC, m–r). Compared with the controls (a, g, m, s), overexpression of APP causes an increased number of Lyso Tracker (LTR, b, h), mCherry‐Atg8a (n), and GFP‐Atg8a (t) puncta, which are suppressed by depletion of *CHIP* or *Atg*, but not by expression of Dcr2 (c–f, i–l, o–r, u–x). (i–iv) Quantification of the number of LTR, mCherry‐Atg8a, and GFP‐Atg8a punctate structures in a–x. Values are shown as mean ± *SEM*. *<0.05, **p < .01, ****p < .0001, ns, not significant. *n* > 15 larvae per genotype. Scale bars, 100 μm

Marked accumulation of autophagosomes and late autophagic vacuoles is found in postmortem brain samples of AD patients (Nixon et al., 2005; Yu et al., 2004), suggesting aberrant autophagy is associated with neurodegeneration (Nixon, 2007; Zare‐Shahabadi et al., 2015), yet it remains elusive whether abnormal autophagy is a cause or consequence of degenerative process in AD. To address this issue, we examined whether overexpression of APP led to autophagy defects in 3rd instar imaginal disks by staining with Lyso Tracker Red, an effective marker of autolysosome. Compared with the controls (Figure 5a,g), Lyso Tracker‐positive puncta are notably increased in corresponding areas of the wing (Figure 5b) or eye disks (Figure 5h) where APP was expressed by *ptc*‐Gal4 or *GMR*‐Gal4, respectively. These data were verified by the accumulation of Atg8a puncta, a widely used markers for autophagic vesicles (Mauvezin, Ayala, Ayala, Braden, Kim, & Neufeld, 2014). Compared with the controls, an increased number of mCherry‐Atg8a or GFP‐Atg8a puncta were detected in 3rd instar larval VNC (Figure 5m,n), adult brains (Figure S8), and eye disks (Figure 5s,t), where APP was specifically expressed by *Appl*‐Gal4 and *GMR*‐Gal4, respectively. Thus, ectopic APP expression is sufficient to trigger aberrant autophagy in both neuronal and non‐neuronal tissues*.*

On the other hand, CHIP has been reported to promote or inhibit autophagy (Arndt et al., 2010; Ferreira et al., 2013; Guo et al., 2015), yet its role in APP‐induced autophagy remains unknown. We found that APP‐induced autophagic vacuole accumulation in the larval VNC, wing, or eye disks was significantly impeded by depletion of *CHIP*, but not expression of Dcr2 (Figure 5c–e,i–k,o–q,u–w and i–iv). As a positive control, APP‐induced autophagy defect was effectively eliminated by knocking down *Atg7* (Figure 5f,l,r,x). Taken together, these results demonstrate that *CHIP* plays an essential role in APP‐induced autophagy abnormality in both neuronal and non‐neuronal tissues.

### 2.8 Blocking autophagy suppresses APP‐induced morphological and behavioral defects

**Figure 6:**
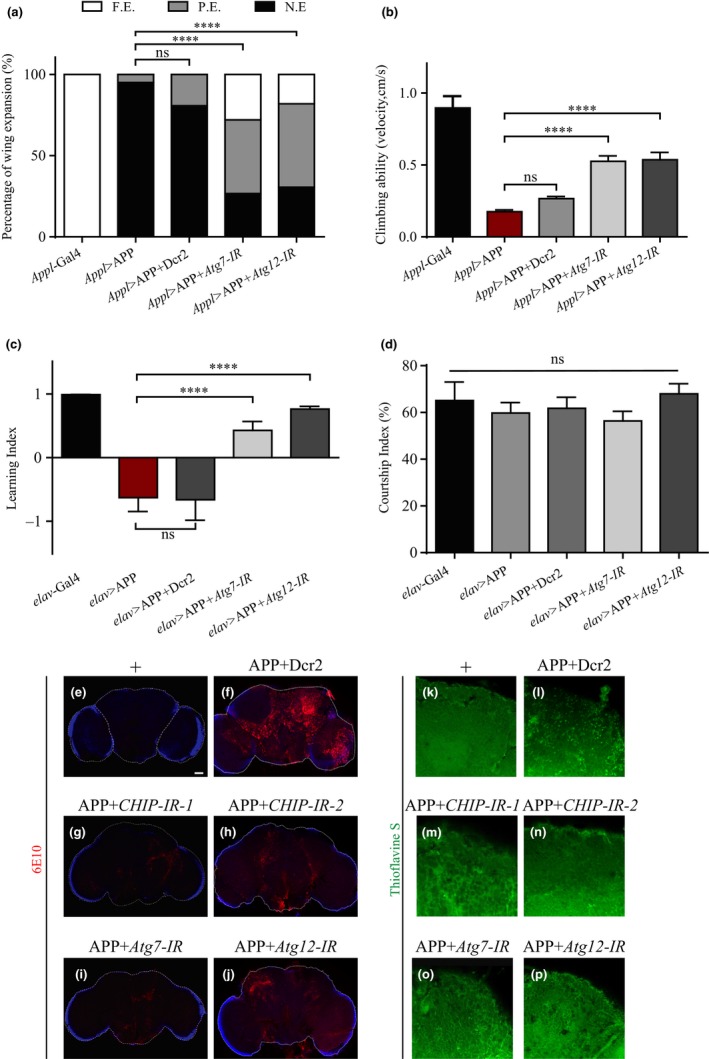
Blocking autophagy impedes APP‐induced pathological functions and Aβ production. (a) Histogram showing the percentage of adult wings with morphological defects in different genotype flies (abbreviation: N.E., no expansion; P.E., partial expansion; F.E., full expansion). Overexpression of APP using *Appl*‐Gal4 produces an infant wing phenotype, which is suppressed partially or fully by knocking down *Atg7* or *Atg12*. *n* > 100 females for each genotype. (b) Histogram showing the climbing abilities of 2‐day‐old female flies with indicated genotypes. Depletion of *Atg7* or *Atg12* suppresses APP‐induced locomotion defect. *n* > 200 females for each genotype. (c) Learning index of 3‐day‐old naive males with indicated genotypes in the courtship suppression assay. Compared with *elav*‐Gal4 controls, overexpression of APP driven by *elav*‐Gal4 displays a learning impairment, which is improved by inhibiting autophagy. (d) Courtship index of matched 3‐day‐old naive males in the initial 10 min of the training phase in the courtship suppression assay. *n* > 30 males for each genotype. (e–j) Confocal images of 2‐day‐old females’ brains stained with Aβ‐specific antibody 6E10. Compared with the controls (e), flies co‐expressing APP with Dcr2 accumulate intensive fluorescence in the central brains (f), which is effectively reduced by knocking down *CHIP* (g, h), *Atg7* (i), or Atg12 (j). (k‐p) Confocal images of 7‐day‐old females’ brains stained with thioflavin S to detect the amyloid deposits. Compared with the controls (k), APP expression results in increased amyloid deposits (l), which are effectively reduced by knocking down *CHIP* (m, n), *Atg7* (o), or Atg12 (p). Scale bars, 50 μm

To investigate whether disordered autophagy contributes to APP‐induced pathological symptoms, we blocked autophagy in APP‐expressing flies by knocking down *Atg7* or *Atg12*, which encodes an essential regulator of autophagosome assembly and is necessary to promote autophagy initiation. As APP overexpression in *Drosophila* triggers pathological defects in morphology, behavior, and cognition, we examined three representative phenotypes—wing expansion, locomotor activity, and learning ability, respectively. We found that APP‐induced wing expansion defect (Figure 6a), climbing disability (Figure 6b), and learning deficit (Figure 6c) were significantly suppressed by blocking autophagy initiation. As a control, blocking autophagy did not affect the normal courtship behavior of APP‐expressing flies (Figure 6d). Thus, aberrant autophagy contributes to APP‐induced morphological, behavioral, and cognitive defects in *Drosophila*.

### 2.9 *CHIP* depletion impedes Aβ production and accumulation in APP‐expressing brains

Autophagy plays a pivotal role in the clearance of cellular waste, including toxic protein aggregates. However, recent studies reveal that blocking autophagy impedes Aβ plaque load in AD mice (Nilsson et al., 2013). Thus, decrease of *CHIP* may alleviate APP‐induced AD‐like symptoms by blocking autophagy‐mediated Aβ production and accumulation. To test this possibility, *Drosophila* brains were immuno‐stained with a specific Aβ antibody. As expected, *Appl*‐Gal4 control flies showed little fluorescence in their brains (Figure 6e), while flies co‐expressing APP with Dcr2 accumulated intense fluorescence throughout the central brains (Figure 6f), suggesting an increased level of Aβ. Depletion of *CHIP* or blocking autophagy by knocking down *Atg7* or *Atg12* significantly suppressed APP‐induced Aβ accumulation (Figure 6g‐j). We also performed the thioflavin S (TS) staining in the adult brains to specifically visualize the amyloid deposits (Chiang, Wang, Wang, Xie, Yau, & Zhong, 2010). Consistent with the antibody staining, we observed TS‐positive deposits in APP‐expressing brains, which were effectively reduced by knocking down *CHIP*, *Atg7,* or *Atg12* (Figure 6k‐p). The decreased Aβ level is not caused by reduced APP expression, since neither APP mRNA nor protein level was significantly changed upon depletion of *CHIP* or *Atg* (Figure S9). Thus, the above results suggest that CHIP promotes autophagy‐mediated Aβ production and accumulation.

## 3 DISCUSSION

In this study, we exploited the powerful strength of *Drosophila* genetics by expressing human APP to recapitulate a number of AD features, including locomotor dysfunction, eye neurodegeneration, longevity shortening, DA neuron loss, choice, and learning deficits. These AD fly models could contribute to identify and characterize factors modulating APP functions in the pathogenesis of AD.

Although AD is characterized by extracellular Aβ aggregates and intraneuronal accumulations of hyperphosphorylated Tau, autophagy deficits likely precede the appearance of these pathological hallmarks (Nixon, 2007; Nixon et al., 2005). Despite a strong correlation between impaired autophagy and AD, the role of autophagy in AD pathogenesis has been controversial. For example, induction of autophagy by rapamycin promotes Aβ clearance, lowers intracellular Aβ level, reduces plaque load, and improves cognition (Caccamo, Majumder, Majumder, Richardson, Strong, & Oddo, 2010). Consistent with these results, loss of autophagy‐initiating Beclin1 increases both intracellular and extracellular Aβ load (Pickford et al., 2008). On the other hand, autophagy deficiency impedes Aβ secretion and reduces extracellular Aβ accumulation and plaque burden in AD mice (Nilsson et al., 2013). Consistently, high brain cholesterol impairs Aβ degradation but stimulates autophagy‐dependent Aβ secretion and amyloid deposit formation (Barbero‐Camps et al., 2018). In this study, we found that blocking autophagy not only reduces Aβ production and accumulation, but also suppresses APP‐induced morphological, behavioral, and cognitive defects. Thus, aberrant autophagy is a consequence of enhanced APP activity and also contributes to APP‐induced pathological symptoms by facilitating Aβ production.

CHIP, a well‐conserved protein with ∼60% amino acid sequence similarity between human and fruit fly (Paul & Ghosh, 2014), is highly expressed in tissues with high metabolic activity and vibrant protein turnover. CHIP is known to function as a protein quality control or modifier of apoptosis. In addition, recent studies have pointed out an alternative function of CHIP modulation of autophagy (Arndt et al., 2010; Ferreira et al., 2013; Guo et al., 2015). However, contradictory results have been reported about the role of CHIP in autophagy. On one hand, CHIP is reported to initiate chaperone‐assisted selective autophagy (CASA) by cooperating with HSP70 and BAG3 to degrade the damaged Z‐disk proteins (Arndt et al., 2010). On the other hand, knockdown CHIP induces autophagosome formation by increasing the PTEN protein level and decreasing the AKT/mTOR activity, suggesting a suppressive effect of CHIP on autophagy (Guo et al., 2015). Additionally, there have been studies focusing on CHIP‐mediated clearance of tau in AD. Petrucelli *et al.* showed that CHIP interacted directly with Hsp70 to induce tau ubiquitination, which may protect against tau aggregation and neurofibrillary degeneration (Petrucelli et al., 2004). However, they also found excess CHIP dramatically increased the accumulation of tau, suggesting that the balance between CHIP and substrate levels may be critical.

In this study, we found that decrease of *CHIP* strikingly suppressed APP‐induced aberrant autophagy, which was indispensable for APP‐induced pathological symptoms. As CHIP is known to regulate other biological processes, for example, apoptosis and protein quality control, it is intriguing to test whether these roles of CHIP contribute to APP’s pathological functions. We found that blocking cell death by expressing Diap1 or P35, or impeding the ubiquitin–proteasome system (UPS) by knocking down Rpn7 or Rpt4 (components of the 19S regulatory subunits), or Prosβ3 (component of the 20S core subunits) failed to suppress APP‐induced Aβ production, DA neuron loss, and wing expansion defect (Figure S10), implying the roles of CHIP in apoptosis and UPS‐mediated protein quality control are not involved in the pathological function of APP.

We are also curious about how autophagy contributes to APP‐induced Aβ production, which results from a sequential cleavage of APP by β‐ and γ‐secretases. To this end, we examined the transcriptional level of β‐secretase (BACE1) and components of γ‐secretase (*Psn*, *Aph‐1*, *nicastrin*). Intriguingly, we found blocking autophagy decreased the mRNA levels of *BACE1* (Figure S11a) and *Psn* (Figure S11b), but not that of *Aph‐1* and *Nicastrin* (Figure S11c,d). These data imply that autophagy may regulate APP cleavage and Aβ production via affecting *BACE1* and *Psn* expression.

Finally, since *Drosophila* encodes an APP‐like protein, APPL, which is also subjected to cleavage by the β‐ and γ‐secretases, we wonder whether the cleavage of APPL is regulated by autophagy. To this end, we performed Western blot assay and found that α‐CTF, produced from the α‐cleavage of APPL, was significantly increased upon blocking autophagy (Figure S12), which is consistent with our observation that *BACE1* and *Psn* expression depends on autophagy (Figure S11a,b).

## 4 EXPERIMENTAL PROCEDURES

### 4.1 Statistical analysis

Unless otherwise noted, one‐way ANOVA followed by Tukey's multiple comparison test was used to determine the statistical significance between multiple genotypes. Specifically, for Figures 1e, 6a, and S10m, Kruskal–Wallis rank sum test followed by Nemenyi multiple comparisons test with Benjamini and Yekutieli's p value adjustment was applied. For Figure 4b, Mantel–Cox log‐rank statistical analysis was used to determine the *p* values of median lifespan between different genotypes. For Figure 4c, Wilcoxon matched‐pairs test was used to determine significance between intragroup courtships toward younger females and older ones. All values are shown as mean ± *SEM*. *p < .05, **p < .01, ***p < .001, ****p < .0001, ns, not significant.

Additional experimental procedures are available in Supporting Information.

## CONFLICT OF INTEREST

The authors declare no conflict of interest.

## AUTHOR CONTRIBUTIONS

L.Z., X.S., and L.X. conceived and designed the experiments. L.Z, F.P, Y.H., W.L., Y.C., J.H., P.R., Y.Z., and E.X. performed the experiments. L.Z., X.S., and. L.X analyzed the data. W.L., F.P., J.H., P.R., Y.S., and X.G. provided technical assistance. Z.L. and L.X. wrote the manuscript.
