# Supplementary material for: CHIP modulates APP‐induced autophagy‐dependent pathological symptoms in Drosophila
Source: Aging Cell. 2019 Nov 28;19(2):e13070. doi: 10.1111/acel.13070 (PMC6996943; doi:10.1111/acel.13070)
Supplement: Supplementary file 1 [file ACEL-19-e13070-s001.docx]

**SUPPORTING INFORMATION**

***CHIP* modulates APP-induced autophagy-dependent pathological symptoms in *Drosophila***

Luming Zhuang^1^, Fei Peng^1^, Yuanyuan Huang^1^, Wenzhe Li^1^, Jiuhong Huang^2^, Yunqiang Chu^1^, Pu Ren^1^, Ying Sun^1^, Yan Zhang^1^, Elleen Xue^3^, Xiaowei Guo^1^, Xiafeng Shen^*,1^ and Lei Xue^*,1^

**Zhuang et al., Figure S1**


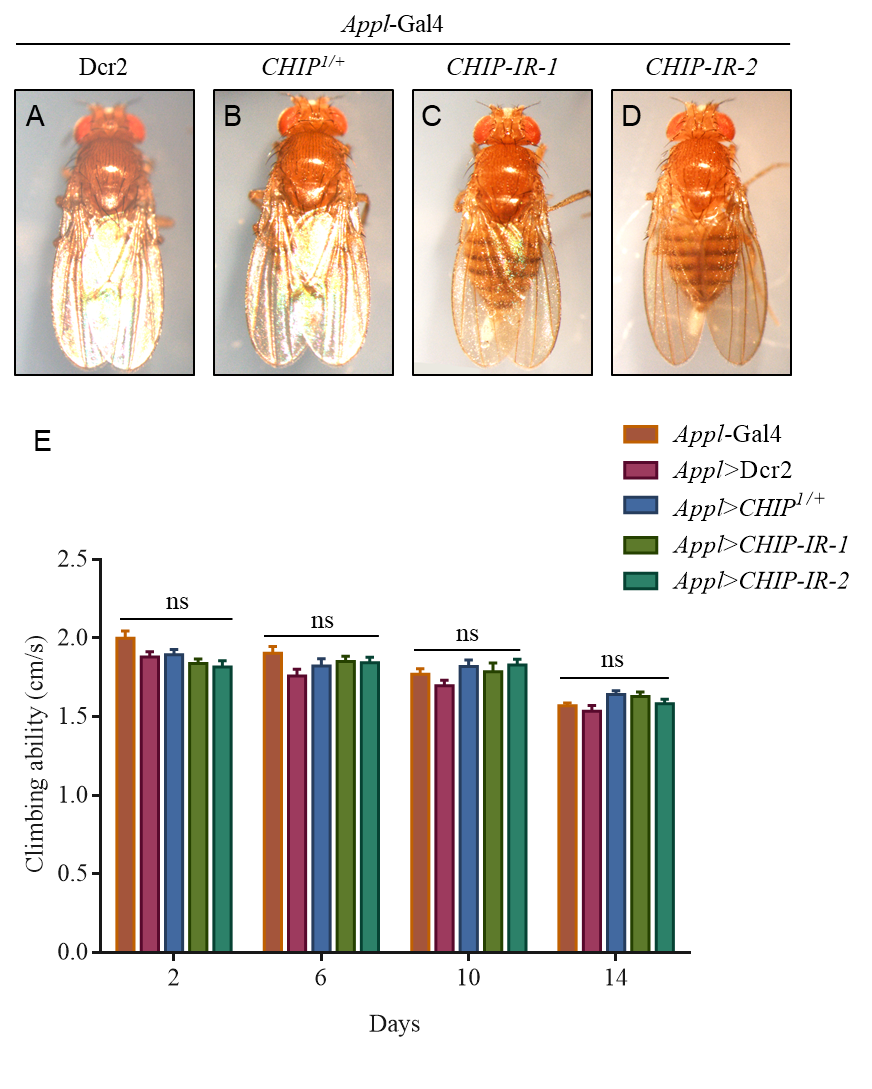


**Figure S1. Depletion of *CHIP* alone does not affect the wing morphology and climbing ability of adult flies.** (A-D) Images showing the wing phenotype of adult females. Flies expressing Dcr2 (A), or heterozygous for *CHIP^1^* (B), or expressing two independent *CHIP*-RNAi (C, D) show normal wing phenotype. (E) Histograms showing longitudinal activity of the female flies with indicated genotypes at different time points. All values are shown as mean ± SEM. ns, not significant. n>100 female flies for each genotype.

**Zhuang et al., Figure S2**

**Figure S2. *CHIP* transcription is not changed upon APP overexpression.** The relative *mRNA* levels of *CHIP* in the adult heads and larval brains were analyzed by qRT-PCR. Compared with the *Appl*-Gal4 controls, the relative *CHIP mRNA* levels in APP-expressing adult heads or larval brains are not significantly changed. n=2 independent biological replicates. All values are shown as mean ± SEM. ns, not significant.

**Zhuang et al., Figure S3**


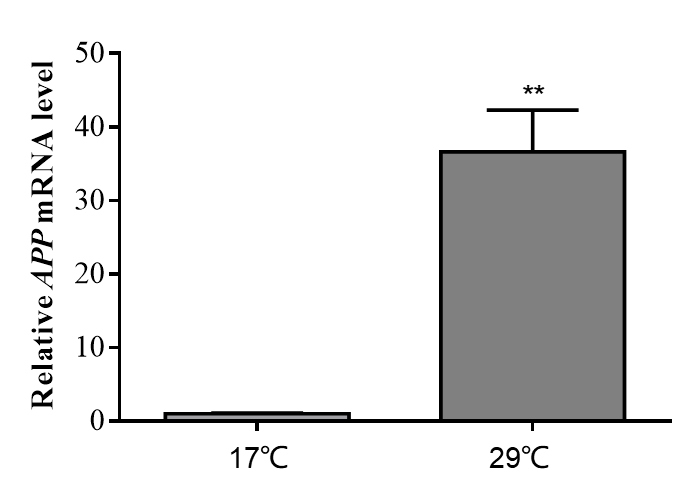


**Figure S3. Temperature dependent expression of APP driven by *Appl*-Gal4.** Due to the temperature dependence of Gal4 activity, APP was barely expressed at 17℃, but was dramatically induced after being shifted to 29℃ for 24 hours. Total mRNA was extracted from the adult heads and qRT-PCR assay was performed. n=3 independent biological replicates. All values are shown as mean ± SEM. **P<0.01.

**Zhuang et al., Figure S4**

**
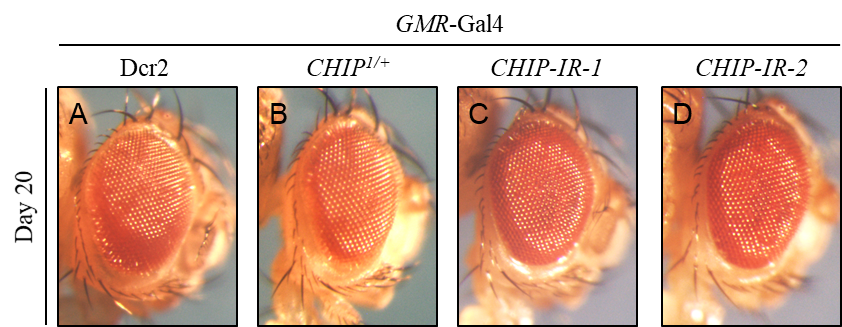
**

**Figure S4. Decrease of *CHIP* exhibits normal eye morphology.** (A-D) Optical microscopic images showing eye phenotype of 20-day-old females. Flies expressing Dcr2 (A), or heterozygous for *CHIP^1^* (B), or expressing two independent *CHIP*-RNAi (C, D) exhibit normal eye phenotype.

**Zhuang et al., Figure S5**

**
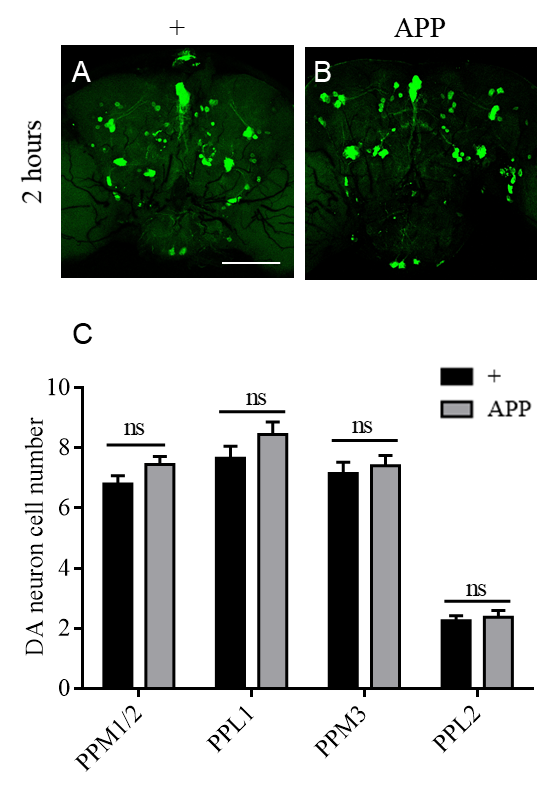
**

**Figure S5. APP-induced DA neuron loss is not a developmental defect.** (A, B) Confocal images of DA neuron clusters in adult posterior brains. Compared with the controls (A), overexpression of APP (B) has no effect on the number of DA neuron in the adult brains at 2 hours after eclosion. (C) Statistical analysis showing the number of GFP-positive cells in different DA neuron clusters. Values are shown as mean ± SEM. ns, not significant. n=15 brains per genotype. Scale bars, 50μm.

**Zhuang et al., Figure S6**


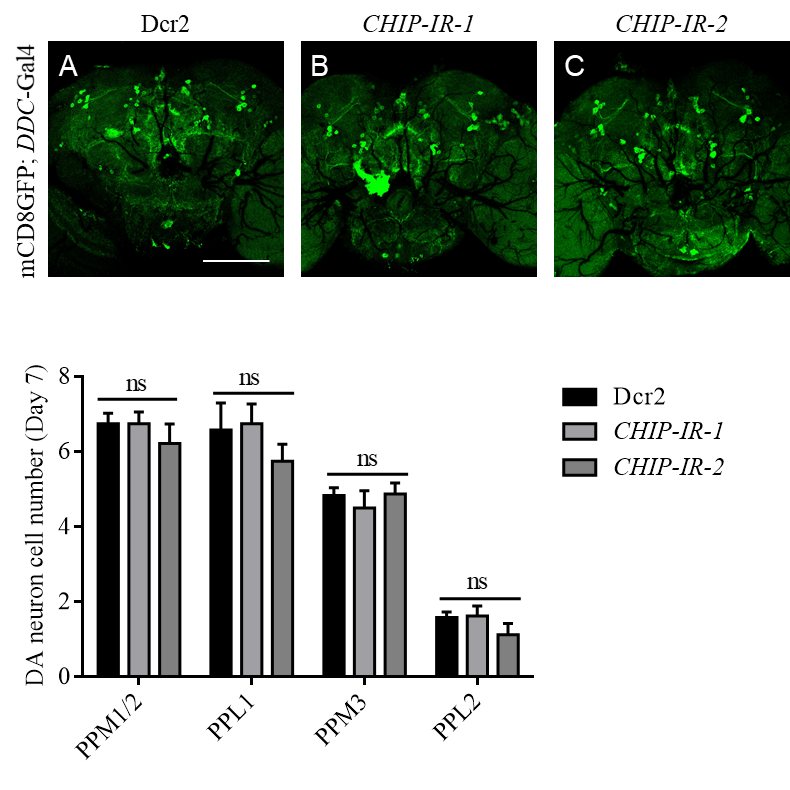


**Figure S6. Decrease of *CHIP* does not affect the number of DA neurons.** (A-C) Confocal images of DA neuron clusters in adult posterior brains. Compared with the controls (A), depletion of *CHIP* does not affect the number of DA neurons in 7 day-old flies (B, C). (D) Statistical analysis showing the number of GFP-positive cells in different DA neuron clusters. Values are shown as mean ± SEM. ns, not significant. n=15 brains per genotype. Scale bars, 50μm.

**Zhuang et al., Figure S7**

**
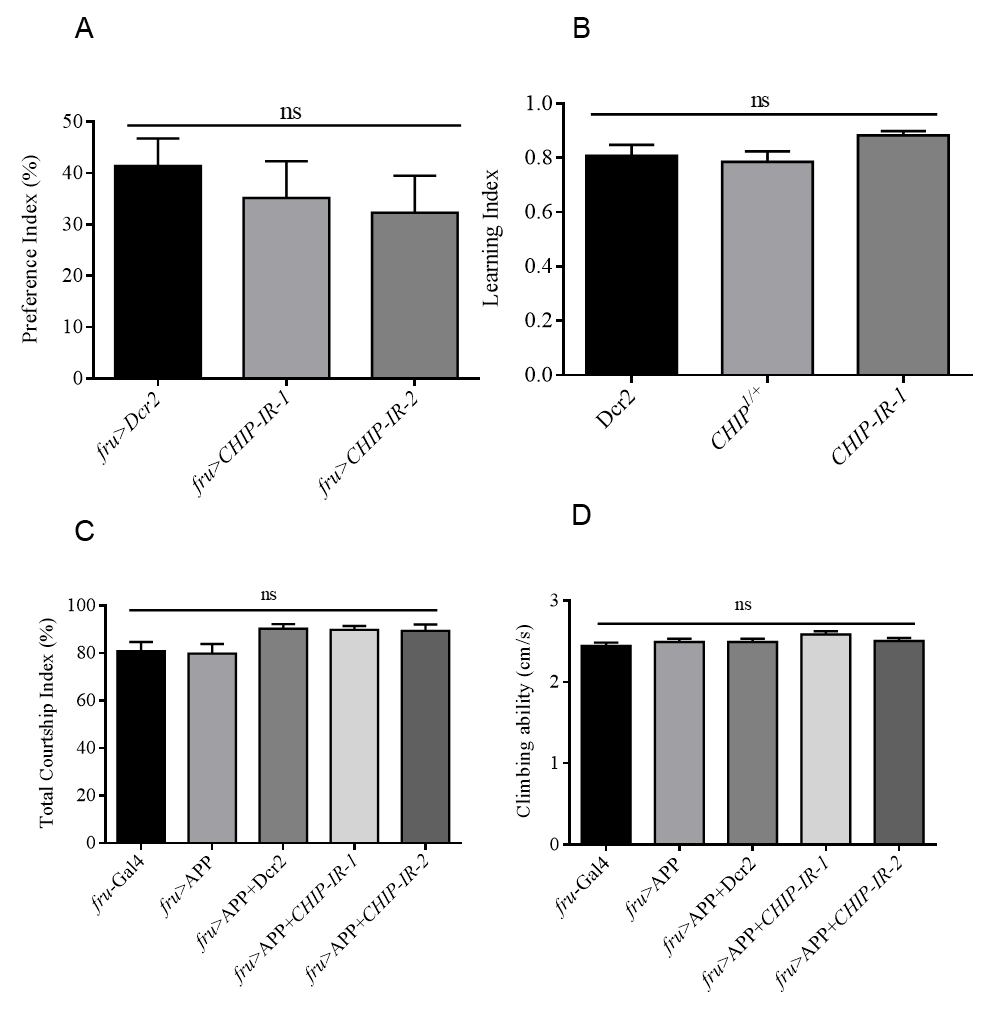
**

**Figure S7. Decrease of *CHIP* does not affect courtship choice and learning ability.** (A) Preference index in the courtship choice assay. 3-day-old naive males expressing Dcr2 or *CHIP-IR* driven by *fru*-Gal4 exhibit a normal courtship preference for younger mates. n>30 males per genotype. (B) Learning index in the courtship suppression assay. 3-day-old naive males expressing Dcr2 or *CHIP-IR* by *elav*-Gal4, or heterozygous for *CHIP^1^* mutation, show normal learning ability. n>30 males per genotype. (C) The total courtship indexes of 3-day-old naive males from the indicated genotypes are not significantly different. n>30 males per genotype. (D) The climbing activities of 3-day-old naive males are not significantly different among the indicated genotypes. n>100 males per genotype. Values are shown as mean ± SEM. ns, not significant.

**Zhuang et al., Figure S8**


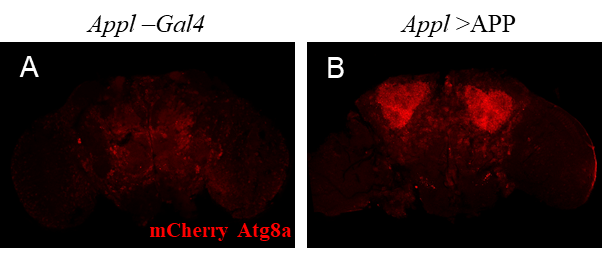


**Figure S8. APP induces increased autophagy in the adult brain.** (A, B) Confocal images of mCherry-Atg8a positive puncta in the adult brain. Compared with *Appl*-Gal4 controls (A), overexpression of APP driven by *Appl*-Gal4 causes an increasing number of mCherry-Atg8a puncta in the adult brain (B).

**Zhuang et al., Figure S9**

**
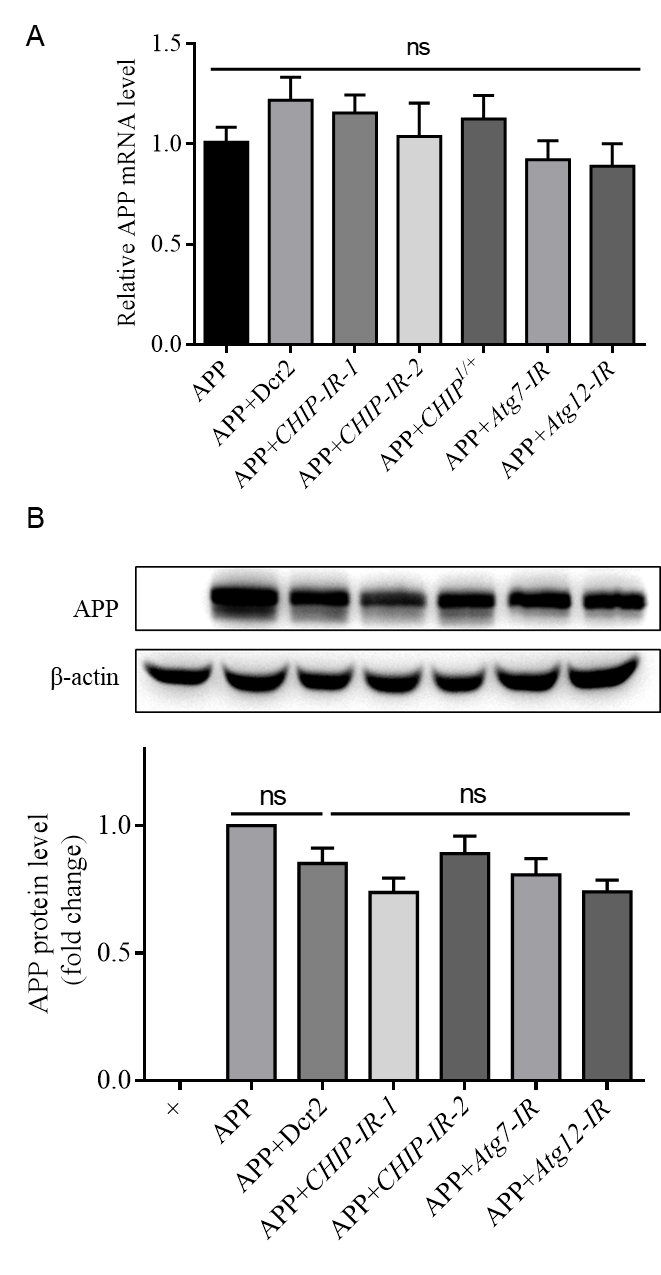
**

**Figure S9. APP expression is not affected by depletion of *CHIP* or *Atg.*** (A) Analyzation of APP mRNA level in the adult heads by qRT-PCR. (B) Quantification of APP protein level in adult heads by western blot. Compared with APP-expressing flies, depletion of *CHIP* or *Atg* has no significant effect on APP mRNA and protein levels. n=2 independent biological replicates. All values are shown as mean ± SEM. ns, not significant.

**Zhuang et al., Figure S10**

**
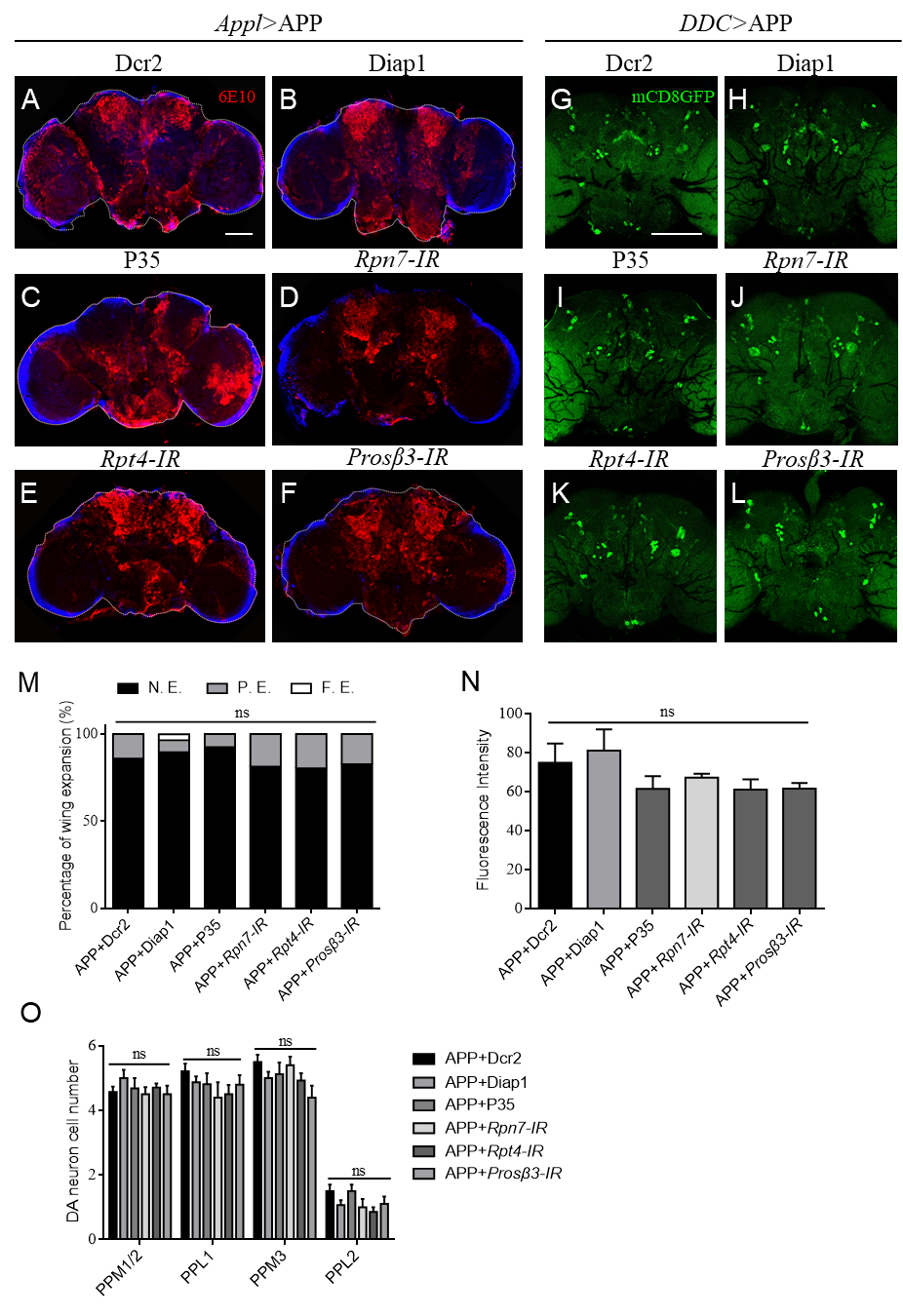
**

**Figure S10. APP-induced pathlogical symptoms are independent of apoptosis and protein quality control.** (A-F) Confocal images of 2-day-old brains stained with Aβ-specific antibody 6E10. Co-overexpression of APP and Dcr2 accumulates 6E10-positive fluorescence signal in the central brains (A), which is not affected by blocking the apoptotic pathway with Diap1 (B) or P35 (C) expression, or the proteasomal activity with depletion of *Rpn7* (D), *Rpt4* (E) or *Prosβ3* (F). (G-L) Confocal images of DA neuron clusters in adult posterior brains. Co-overexpression of APP and Dcr2 results in a reduction of cell number in most DA neuron clusters from 2-day-old adult brains (G), which is affected by blocking the apoptotic pathway (H, I) or the proteasomal activity (J, K, L). (M) Histogram showing the percentage of adult wing phenotypes in different genotypes. The number of female flies tested for each genotype is n>50. (N) Quantification of fluorescent intensity in the central brains of adult flies. n=15 brains per genotype. (O) Statistical analysis showing the number of GFP-positive cells in different DA neuron clusters. Values are shown as mean ± SEM. ns, not significant. Scale bars, 50μm.

**Zhuang et al., Figure S11**

**
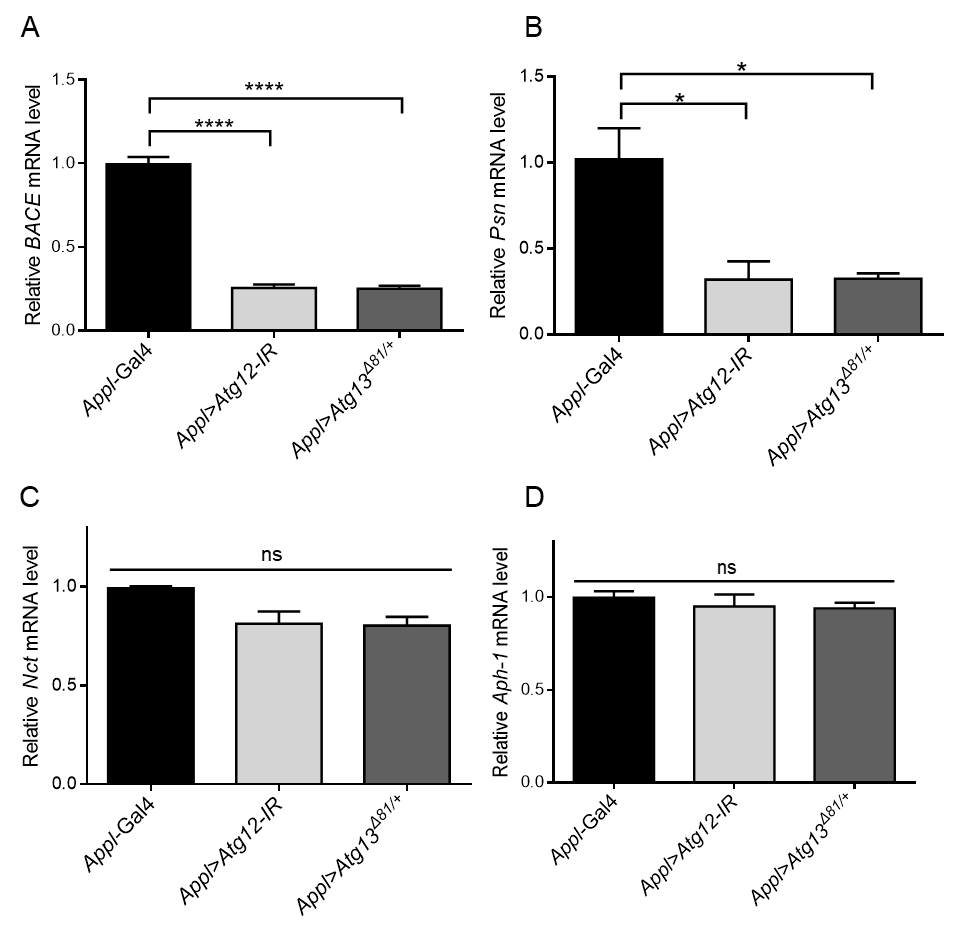
**

**Figure S11. Autophagy regulates the transcription of *BACE* and *Psn.*** (A-D) The relative mRNA levels of *BACE*, *Psn*, *Nicastrin* and *Aph-1* in the adult heads are measured by qRT-PCR. Compared with the controls, impaired autophagy by *Atg12-IR* or *Atg13^Δ81/+^* reduces the mRNA levels of *BACE1* (A) and *Psn* (B), but has no influence on the mRNA levels of *Nicastrin* (C) and *Aph-1* (D)*.* n=3 independent biological replicates. All values are shown as mean ± SEM. *<0.05, ****P<0.0001, ns, not significant.

**Zhuang et al., Figure S12**


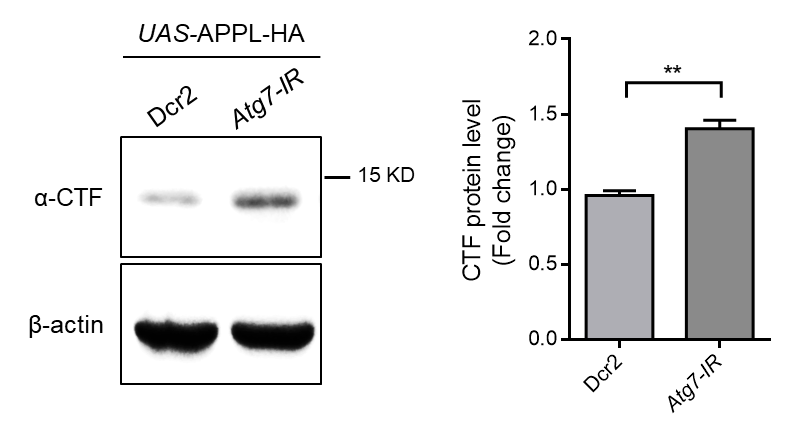


**Figure S12. Autophagy regulates the cleavage of APPL.** Western Blots of adult head homogenates using an HA-Tag antibody against the C-terminal APPL fragment. Western Blot from flies co-expressing APPL-HA and Dcr2 by *GMR*-Gal4 shows the α-CTF fragment of ~15 kD, the predominant α-cleavage product of APPL (lanes 1). Blocking autophagy by expressing *Atg7-IR* (lanes 2) increases the production of the α-CTF fragment. n=3 independent biological replicates. All values are shown as mean ± SEM. **P<0.01.

**Detailed Genotypes for All Figures**

**Figure 1**

*Appl*-Gal4/+

*Appl-*Gal4/+; *UAS*-APP/+

*Appl-*Gal4/+; *UAS*-APP/+; *UAS*-Dcr2/+

*Appl-*Gal4/+; *UAS*-APP/+; *CHIP^1^/+*

*Appl-*Gal4/+; *UAS*-APP/+; *UAS-CHIP-IR-1*/+

*Appl-*Gal4/+; *UAS*-APP/+; *UAS-CHIP-IR-2*/+

**Figure 2**

(A, B, K, L) *GMR*-Gal4/+

(C, D, M, N) *GMR*-Gal4 *UAS*-APP/*UAS*-APP

(E, F, O, P) *GMR*-Gal4 *UAS*-APP/*UAS*-APP; *UAS*-Dcr2/+

(G, H, Q, R) *GMR*-Gal4 *UAS*-APP/*UAS*-APP; *UAS-CHIP-IR-1*/+

(I, J, S, T) *GMR*-Gal4 *UAS*-APP/*UAS*-APP; *UAS-CHIP-IR-2*/+

**Figure 3**

(A, B) *UAS*-mCD8GFP/+; *DDC*-Gal4/+

(C, D) *UAS*-mCD8GFP/*UAS*-APP; *DDC*-Gal4/+

(E, F) *UAS*-mCD8GFP/*UAS*-APP; *DDC*-Gal4/*UAS*-Dcr2

(G, H) *UAS*-mCD8GFP/*UAS*-APP; *DDC*-Gal4/*UAS*-*CHIP-IR-1*

(I, J) *UAS*-mCD8GFP/*UAS*-APP; *DDC*-Gal4/*UAS*-*CHIP-IR-2*

**Figure 4**

(A, B) *Appl*-Gal4/+

*Appl-*Gal4/+; *UAS-CHIP-IR-1*/+

*Appl-*Gal4/+; *UAS-CHIP-IR-2*/+

*Appl-*Gal4/+; *UAS*-APP/+

*Appl-*Gal4/+; *UAS*-APP/+; *UAS*-Dcr2/+

*Appl-*Gal4/+; *UAS*-APP/+; *UAS-CHIP-IR-1*/+

*Appl-*Gal4/+; *UAS*-APP/+; *UAS-CHIP-IR-2*/+

(C, D) *fru*-Gal4/+

*UAS*-APP/+; *fru-*Gal4/+

*UAS*-APP/+; *fru-*Gal4/*UAS*-Dcr2

*UAS*-APP/+; *fru-*Gal4/*UAS-CHIP-IR-1*/+

*UAS*-APP/+; *fru-*Gal4/*UAS-CHIP-IR-2*/+

(E, F) *elav*-Gal4/+

*UAS*-APP/+; *elav-*Gal4

*UAS*-APP/+*; elav-*Gal4/*CHIP^1^*

*UAS*-APP/+; *elav-*Gal4/*UAS-CHIP-IR-1*

**Figure 5**

(A) *ptc*-Gal4/+

(B) *ptc*-Gal4/*UAS*-APP

(C) *ptc*-Gal4/ *UAS*-APP; *UAS-*Dcr2/+

(D) *ptc*-Gal4/ *UAS*-APP; *UAS-CHIP-IR-1*/+

(E) *ptc*-Gal4/ *UAS*-APP; *UAS-CHIP-IR-2*/+

(F) *ptc*-Gal4/ *UAS*-APP; *UAS-Atg13^Δ81^*/+

(H) *UAS*-APP/+; *GMR*-Gal4/*+*

(I) *UAS*-APP/+; *GMR*-Gal4/*UAS-*Dcr2

(J) *UAS*-APP/+; *GMR*-Gal4/*UAS-CHIP-IR-1*

(K) *UAS*-APP/+; *GMR*-Gal4/*UAS-CHIP-IR-2*

(L) *UAS*-APP/+; *GMR*-Gal4/*UAS-Atg13^Δ81^*

(M) *Appl*-Gal4/+; *UAS*-mCherry-Atg8a/+

(N) *Appl*-Gal4/+; *UAS*-mCherry-Atg8a/*UAS*-APP

(O) *Appl*-Gal4/+; *UAS*-mCherry-Atg8a/*UAS*-APP; *UAS*-Dcr2/+

(P) *Appl*-Gal4/+; *UAS*-mCherry-Atg8a/*UAS*-APP; *UAS-CHIP-IR-1*/+

(Q) *Appl*-Gal4/+; *UAS*-mCherry-Atg8a/*UAS*-APP; *UAS-CHIP-IR-2*/+

(R) *Appl*-Gal4/+; *UAS*-mCherry-Atg8a/*UAS*-APP; *UAS-Atg7-IR*/+

(S) *GMR*-Gal4/+; *UAS*-GFP-Atg8a/+

(T) *GMR*-Gal4 *UAS*-APP/*UAS*-APP; *UAS*-GFP-Atg8a/+

(U) *GMR*-Gal4 *UAS*-APP/*UAS*-APP; *UAS*-GFP-Atg8a/*UAS*-Dcr2

(V) *GMR*-Gal4 *UAS*-APP/*UAS*-APP; *UAS*-GFP-Atg8a/*UAS*-*CHIP-IR-1*

(W) *GMR*-Gal4 *UAS*-APP/*UAS*-APP; *UAS*-GFP-Atg8a/*UAS*-*CHIP-IR-2*

(X) *GMR*-Gal4 *UAS*-APP/*UAS*-APP; *UAS*-GFP-Atg8a/*UAS*-*Atg7-IR*

**Figure 6**

(A, B) *Appl*-Gal4/+

*Appl-*Gal4/+; *UAS*-APP/+

*Appl-*Gal4/+; *UAS*-APP/+; *UAS*-Dcr2/+

*Appl-*Gal4/+; *UAS*-APP/+; *UAS-Atg7-IR/+*

*Appl-*Gal4/+; *UAS*-APP/+; *UAS-Atg12-IR/+*

(C, D) *elav*-Gal4/+

*UAS*-APP/+; *elav-*Gal4/+

*UAS*-APP/+; *elav-*Gal4/*UAS*-Dcr2

*UAS*-APP/+; *elav-*Gal4/*UAS*- *Atg7-IR*

*UAS*-APP/+; *elav-*Gal4/*UAS*- *Atg12-IR*

(E, K) *Appl*-Gal4/+

(F, L) *Appl-*Gal4/+; *UAS*-APP/+; *UAS*-Dcr2/+

(G, M) *Appl-*Gal4/+; *UAS*-APP/+; *UAS-CHIP-IR-1*/+

(H, N) *Appl-*Gal4/+; *UAS*-APP/+; *UAS-CHIP-IR-2*/+

(I, O) *Appl-*Gal4/+; *UAS*-APP/+; *UAS-Atg7-IR*/+

(J, P) *Appl-*Gal4/+; *UAS*-APP/+; *UAS-Atg12-IR*/+

**Figure S1**

(A) *Appl*-Gal4/+; *UAS-*Dcr2/+

(B) *Appl*-Gal4/+; *CHIP^1^*/+

(C) *Appl-*Gal4/+; *UAS-CHIP-IR-1*/+

(D) *Appl-*Gal4/+; *UAS-CHIP-IR-2*/+

**Figure S2**

*Appl*-Gal4/+

*Appl-*Gal4/+; *UAS*-APP/+

**Figure S3**

*Appl-*Gal4/+; *UAS*-APP/+

**Figure S4**

(A) *GMR*-Gal4/+; *UAS-*Dcr2/+

(B) *GMR*-Gal4/+; *CHIP^1^*/+

(C) *GMR-*Gal4/+; *UAS-CHIP-IR-1*/+

(D) *GMR-*Gal4/+; *UAS-CHIP-IR-2*/+

**Figure S5**

(A) *UAS*-mCD8GFP/+; *DDC*-Gal4/+

(B) *UAS*-mCD8GFP/*UAS*-APP; *DDC*-Gal4/+

**Figure S6**

(A) *UAS*-mCD8GFP/+; *DDC*-Gal4/*UAS*-Dcr2

(B) *UAS*-mCD8GFP/+; *DDC*-Gal4/ *UAS-CHIP-IR-1*

(C) *UAS*-mCD8GFP/+; *DDC*-Gal4/ *UAS-CHIP-IR-2*

**Figure S7**

(A) *fru-*Gal4/*UAS*-Dcr2

*fru-*Gal4/*UAS-CHIP-IR-1*

*fru-*Gal4/*UAS-CHIP-IR-2*

(B) *elav*-Gal4/*UAS*-Dcr2

*elav-*Gal4/*CHIP^1^*

*elav-*Gal4/*UAS-CHIP-IR-1*

(C, D) *fru*-Gal4/+

*UAS*-APP/+; *fru-*Gal4/+

*UAS*-APP/+; *fru-*Gal4/*UAS*-Dcr2

*UAS*-APP/+; *fru-*Gal4/*UAS-CHIP-IR-1*/+

*UAS*-APP/+; *fru-*Gal4/*UAS-CHIP-IR-2*/+

**Figure S8**

(A) *Appl*-Gal4/+; *UAS*-mCherry-Atg8a/+

(B) *Appl*-Gal4/+; *UAS*-mCherry-Atg8a/*UAS*-APP

**Figure S9**

*Appl*-Gal4/+

*Appl-*Gal4/+; *UAS*-APP/+

*Appl-*Gal4/+; *UAS*-APP/+; *UAS*-Dcr2/+

*Appl-*Gal4/+; *UAS*-APP/+; *UAS-CHIP-IR-1*/+

*Appl-*Gal4/+; *UAS*-APP/+; *UAS-CHIP-IR-2*/+

*Appl-*Gal4/+; *UAS*-APP/+; *UAS-CHIP^1^/+*

*Appl-*Gal4/+; *UAS*-APP/+; *UAS-Atg7-IR*/+

*Appl-*Gal4/+; *UAS*-APP/+; *UAS-Atg12-IR*/+

**Figure S10**

(A) *Appl-*Gal4/+; *UAS*-APP/+; *UAS*-Dcr2/+

(B) *Appl-*Gal4/+; *UAS*-APP/+; *UAS*-Diap1/+

(C) *Appl-*Gal4/+; *UAS*-APP/+; *UAS*-P35/+

(D) *Appl-*Gal4/+; *UAS*-APP/+; *UAS*-*Rpn7-IR*/+

(E) *Appl-*Gal4/+; *UAS*-APP/+; *UAS*-*Rpt4-IR*/+

(F) *Appl-*Gal4/+; *UAS*-APP/+; *UAS*-*Prosβ3-IR*/+

(G) *UAS*-mCD8GFP/*UAS*-APP; *DDC*-Gal4/*UAS*-Dcr2

(H) *UAS*-mCD8GFP/*UAS*-APP; *DDC*-Gal4/*UAS*-Diap1

(I) *UAS*-mCD8GFP/*UAS*-APP; *DDC*-Gal4/*UAS*-P35

(J) *UAS*-mCD8GFP/*UAS*-APP; *DDC*-Gal4/*UAS*-*Rpn7-IR*

(K) *UAS*-mCD8GFP/*UAS*-APP; *DDC*-Gal4/*UAS*-*Rpt4-I*

(L) *UAS*-mCD8GFP/*UAS*-APP; *DDC*-Gal4/*UAS*-*Prosβ3-IR*

**Figure S11**

*Appl-*Gal4/*+*

*Appl-*Gal4/+; *UAS-Atg12-IR/+*

*Appl-*Gal4/+; *UAS-Atg13^Δ81^/+*

**Figure S12**

*GMR*-Gal4/ *UAS-*APPL-HA; *UAS*-Dcr2/+

*GMR*-Gal4/ *UAS-*APPL-HA; *UAS*-*Atg7-IR*/+

**Supplemental Experimental Procedures**

**Fly Strains**

Unless otherwise indicated, all flies were reared on a cornmeal and agar medium according to standard protocols at 25℃, and the data are depicted from female flies. The following fly strains were used in this study: *UAS*-APP (Peng, Zhao et al. 2015), *Appl*-Gal4 (Wang, Wang et al. 2014), *GMR*-Gal4 (Li, Li et al. 2012), *frutless*-Gal4 (Hu, Han et al. 2014) were previously described. *elav*-Gal4 (8760), *hh*-Gal4 (Ma, Guo et al. 2018), *UAS-CHIP-IR-1* (34017), *UAS-CHIP-IR-2* (33938), *UAS-Atg7-IR* (27707), *UAS-Atg12-IR* (27552), *UAS*-Diap1 (6657), *UAS*-P35 were obtained from the Bloomington *Drosophila* Stock Center. *UAS-Rpn7-IR* (THU0633), *UAS-Rpt4-IR* (THU1063), *UAS-Prosβ3-IR* (THU0709) were obtained from the Tsing Hua Fly Center. *DDC*-Gal4 (Song, He et al. 2017) was a gift from professor Margaret S. Ho (School of Life Science and Technology, [ShanghaiTech University](https://www.researchgate.net/institution/ShanghaiTech_University)), *CHIP^1^* (Chen, Xue et al. 2017) was kindly provided by professor Ranhui Duan (The State Key Laboratory of Medical Genetics, School of Life Sciences, Central South University), *Atg13^Δ81^* was a gift from Tor Erik Rusten (Centre for Cancer Biomedicine, University of Oslo). *UAS*-GFP-Atg8a and *UAS*-mCherry-Atg8a were gifts from professor Jingnan Liu (Shanghai Institutes for Biological Sciences, Chinese Academy of Sciences).

**qRT-PCR**

The knockdown efficiency of the RNAi lines and the relative mRNA level of genes were determined by extracting total RNA from about 50 adult heads or 30 larval brains, followed by qRT-PCR. Total RNA was extracted from collecting adult heads or larval brains using TRIzol Reagent according to the manufacturer’s instructions (Invitrogen). cDNA was synthesized using Prime Script RT Master mix (Takara), and qRT-PCR was performed using SYBR Green Master Mix on a Stratagene Mx3000P system (Agilent). To compare the gene expression in different samples, *rp49* was used as an internal reference gene. Three biological replicate samples were analyzed. The following primer sequences were used for RT-PCR:

*Rp49*-FW: 5’-CTGCTCATGCAGAACCGCGT-3’

*Rp49*-PV: 5’-GGACCGACAGCTGCTTGGCG-3’

*CHIP*-FW: 5’-TCAAAGGCCATCATAAAGAACCC-3’

*CHIP*-PV: 5’-TCTTCAGTTTCAGGTTGCAGAG-3’

*APP*-FW: 5’- GCTGAACCCCAGATTGCCATG-3’

*APP*-PV: 5’- GCCTTCCTTGGTATCAATGCAG-3’

*BACE1*-FW: 5’-AGGAGCAGAACTTTGTGAAGAC-3’

*BACE1*-PV: 5’-AAGCCATATTCATCGAGTTGGAC-3’

*Psn*-FW: 5’-CAACAGCAGCGGAACAACTAC-3’

*Psn*-PV: 5’-CCACAAGGTTCACGCATCAC-3’

*Nct*-FW: 5’-AAGATGTACGAGCCCATTGGA-3’

*Nct*-PV: 5’-GGAGTAGGTTGAGGAACAGCC-3’

*Aph-1*-FW: 5’-ACATCCTGGCCTATGTCTCC-3’

*Aph-1*-PV: 5’-CCGGGACCACTCATATCAGC-3’

*Appl*-FW: 5’- CATTGAATGCGGCTAAGTGCAAGG-3’

*Appl*-PV: 5’- GGTCGAACAGACAGCCCTCG-3’

**Climbing and longevity assay**

Climbing assay was performed as previous described (Song, He et al. 2017). In brief, APP-expressing flies were raised at 17°C throughout the larva and pupa stages, then shifted to 29°C after eclosion, and were tested the climb ability every 4 days. About 30 flies were collected in a cylinder (height: 15 cm, inner diameter: 2.5 cm) and were tapped to the bottom of the vial. After 5 seconds, a picture was taken and the average climbing height was measured by fly detection software (provided by professor Fude Huang, Shanghai Advanced Research Institute, Chinese Academy of Sciences). The velocity was calculated by dividing the height (cm) by the time (s). We defined a performance index (PI= velocity_aged_/velocity_young_) to directly evaluate the relative decline of the locomotor ability between aged and young flies in a climbing assay. At least 200 flies were analyzed per genotype.

Longevity assay was carried out as previously described (Peng, Zhao et al. 2015). 25~30 female flies per vial, 8 vials per group were tested. Flies were transferred to fresh medium every 2 days and lost flies were recorded. The proportion of surviving flies in percentage was calculated at each time point. At least 200 flies were tested for each genotype.

**Courtship choice assay**

The assay was performed as described (Hu, Han et al. 2014). Flies were raised at 25°C and 60%~70% humidity in a 12h light-dark cycle. Naive male and virgin female flies were collected after eclosion. A naive male was paired with 2 younger (3-day old) wild type virgin females and 2 older (30-day old) ones in a round observation chamber. The courtship performance was recorded for 10 minutes with a digital video camera (Sony) and analyzed with Noldus Etho Vision XT software (Noldus Information Technology). The courtship index (CI) was calculated as the percentage of time that a male courted the females during a 10-minute period, while CIy and CIo represent the CI towards the younger and older females, respectively. Hence, CI= CIy + CIo. The preference index (PI) was defined as (CIy - CIo)/(CIy + CIo). At least 30 male flies were tested for each genotype.

**Courtship suppression assay**

The procedures were performed as described (Pan, Xie et al. 2015). A female fly was pre-mated by pairing with three wild type males in a vial for 15 hours. For training, individual male was placed in a courtship chamber with a single pre-mated female for 1 hour, and their courtship performance during the initial 10 minutes was recorded by a digital video camera. After training, each male was allowed to recover for 2 minutes, and the courtship activity towards a fresh virgin female was examined within 10 minutes.

**Immunohistochemistry**

The adult brains were collected and dissected in 0.3% PBST and fixed with 4% paraformaldehyde for 30 minutes, washed with PBST (containing 0.3% Tween20) three times before immunofluorescence staining at 4℃ for overnight. For immunostaining, mouse anti-Aβ antibody 6E10 (1:200, SIG-39320, Covance) was used as the primary antibody, and anti-mouse Cy3 (1:1000, Jackson Immuno Research) as the secondary antibody for incubating tissues 2 hours at room temperature. Finally, brains were mounted with DAPI. Images of adult brains were acquired using a Zeiss LSM880 confocal microscope with a 10X objective. At least 15 brains were analyzed per genotype.

**LysoTracker Red staining**

Imaginal discs dissected from third instar larvae were collected in cold PBS and incubated with LysoTracker Red (1:20000, Beyotime) at 37℃ for 15 minutes, washed with PBS three times prior to imaging. At least 15 larvae were dissected per genotype.

**Thioflavin staining**

Thioflavin staining was performed as described (Fay, Fluet et al. 1998). Adult brains were collected and dissected in 0.3% PBST, fixed in 4% paraformaldehyde for 24 hours at 4°C, then permeabilized by incubation in permeabilization solution for 24 hours at 37°C. Permeabilized tissues were transferred to PBST, stained by 0.125% Thioflavin S (Sigma) in 50% ethanol for 2 minutes, washed for 2 minutes in 50% ethanol, and returned to PBST before being mounted for microscopy.

**Fluorescence Intensity calculation**

Only the central area of the brain (exclude the optic lobe) was used for measurement. All the images were taken at the same resolution, same size in pixels at the same scale, with fixed brightness and contrast. The fluorescence intensity was calculated by adding all the green channel value (0-255, RGB model jpg image) and divided by the total pixel number.

**Western blot**

Lysates from 200 adult heads were diluted in SDS sample buffer, separated by 10% SDS gels, and transferred to PVDF membranes (Millipore). The membranes were blocked with 5% nonfat dry milk and blotted with the 6E10 antibody (SIG-39320, 1:1000), anti-HA rabbit antibody (AT0024, CMCTAG, 1:2000) or anti-β-actin antibody (AB0061, Abways Technology, 1:2000). Two biological replicate samples were performed for western blot assay. Data were analyzed with software ImageJ.

**Supplemental Reference**

Chen, J., J. Xue, J. Ruan, J. Zhao, B. Tang and R. Duan (2017). "Drosophila CHIP protects against mitochondrial dysfunction by acting downstream of Pink1 in parallel with Parkin." FASEB J **31**(12): 5234-5245.

Fay, D. S., A. Fluet, C. J. Johnson and C. D. Link (1998). "In vivo aggregation of beta-amyloid peptide variants." J Neurochem **71**(4): 1616-1625.

Hu, Y., Y. Han, X. Wang and L. Xue (2014). "Aging-related neurodegeneration eliminates male courtship choice in Drosophila." Neurobiol Aging **35**(9): 2174-2178.

Li, W. Z., S. L. Li, H. Y. Zheng, S. P. Zhang and L. Xue (2012). "A broad expression profile of the GMR-GAL4 driver in Drosophila melanogaster." Genet Mol Res **11**(3): 1997-2002.

Ma, X. J., X. W. Guo, H. E. Richardson, T. Xu and L. Xue (2018). "POSH regulates Hippo signaling through ubiquitin-mediated expanded degradation." Proceedings of the National Academy of Sciences of the United States of America **115**(9): 2150-2155.

Pan, L., W. Xie, K. L. Li, Z. Yang, J. Xu, W. Zhang, L. P. Liu, X. Ren, Z. He, J. Wu, J. Sun, H. M. Wei, D. Wang, W. Xie, W. Li, J. Q. Ni and F. L. Sun (2015). "Heterochromatin remodeling by CDK12 contributes to learning in Drosophila." Proc Natl Acad Sci U S A **112**(45): 13988-13993.

Peng, F., Y. Zhao, X. Huang, C. Chen, L. Sun, L. Zhuang and L. Xue (2015). "Loss of Polo ameliorates APP-induced Alzheimer's disease-like symptoms in Drosophila." Sci Rep **5**: 16816.

Song, L., Y. He, J. Ou, Y. Zhao, R. Li, J. Cheng, C.-H. Lin and M. S. Ho (2017). "Auxilin Underlies Progressive Locomotor Deficits and Dopaminergic Neuron Loss in a Drosophila Model of Parkinson’s Disease." Cell Reports **18**(5): 1132-1143.

Wang, X., Z. Wang, Y. Chen, X. Huang, Y. Hu, R. Zhang, M. S. Ho and L. Xue (2014). "FoxO mediates APP-induced AICD-dependent cell death." Cell Death Dis **5**: e1233.
